# Iron Deficiency Anemia: An Unexpected Cause of an Acute Occipital Lobe Stroke in an Otherwise Healthy Young Woman

**DOI:** 10.7759/cureus.7852

**Published:** 2020-04-27

**Authors:** Qian Zhang, Khine S Shan, Conor O'Sullivan, Travis Nace

**Affiliations:** 1 Internal Medicine, Abington Hospital-Jefferson Health, Abington, USA; 2 Internal Medicine, University of Maryland Medical Center, Baltimore, USA; 3 Library Science, Abington Hospital-Jefferson Health, Abington, USA

**Keywords:** acute cva, iron deficiency anemia, stroke, iron

## Abstract

A 29-year-old caucasian woman who presented to the hospital with an acute onset of right eye visual disturbance and headache was found to have an acute left occipital lobe infarction. Past medical history was significant for iron deficiency anemia (IDA) secondary to menorrhagia. Her initial hemoglobin level was 7.8 G/DL, and her symptoms improved after iron and blood transfusions. Hypercoagulable studies were completed in the outpatient setting, and the results were unremarkable. Her acute stroke was most likely related to IDA as she had low cardiovascular risk factors along with a negative complete stroke workup.

## Introduction

Ischemic cerebrovascular accident (CVA) or stroke can be caused by either thrombotic, embolic, or hemodynamic events that lead to interruption of blood supply to the brain and subsequent loss of brain function. Approximately 25% of the cases occur in patients less than 65 years old, despite the majority of them occurring in the elderly population [1]. The most common causes of strokes are hypertension and atherosclerosis. Secondary etiologies may include cardioembolic diseases, hypercoagulability states, connective tissue disorders, dissections, arteriovenous malformations (AVM), systemic hypoperfusion, oral contraceptive use, or substance abuse [2, 3]. Furthermore, iron deficiency anemia (IDA) is a very rare etiology of strokes in adults even though it is known to be associated with strokes in the pediatric population [3, 4]. IDA has a prevalence of 2-4% in non-pregnant women ranging from 12-49 years old and a 2-5% prevalence in men and postmenopausal women [3]. IDA is characterized by microcytic hypochromic erythropoiesis with low serum iron and ferritin with an elevation of the total iron-binding capacity [1]. IDA may be caused by poor dietary intake, poor iron absorption, or hemorrhage. IDA may lead to reversible focal neurological deficits, as seen in other metabolic disorders such as hypoglycemia or uremic encephalopathy [5]. However, IDA is often not considered as a risk factor for ischemic stroke as there are very few reported case reports and research studies. Interestingly, our case describes a young woman with an acute left occipital stroke associated with IDA whose symptoms improved subsequently with blood and iron transfusion.

## Case presentation

Our patient is a 29-year-old caucasian, non-smoker woman who initially presented to the hospital due to a sudden onset of blurry vision of the right eye. Her past medical history was significant for obesity status post gastric sleeve surgery, menorrhagia, and iron deficiency anemia. She had a long history of iron deficiency anemia due to menorrhagia since she was 12 years old and had sleeve gastrectomy five years ago. Furthermore, she was treated with oral and intravenous (IV) iron infusion during her pregnancy to control her anemia. Over the past few months, she had been experiencing heavy periods lasting around six days that soaked through multiple pads and tampons a day. She was offered an intrauterine device (IUD) by her gynecologist but declined and elected to use an estrogen and progestin transdermal patch for contraception.

**Figure 1:**
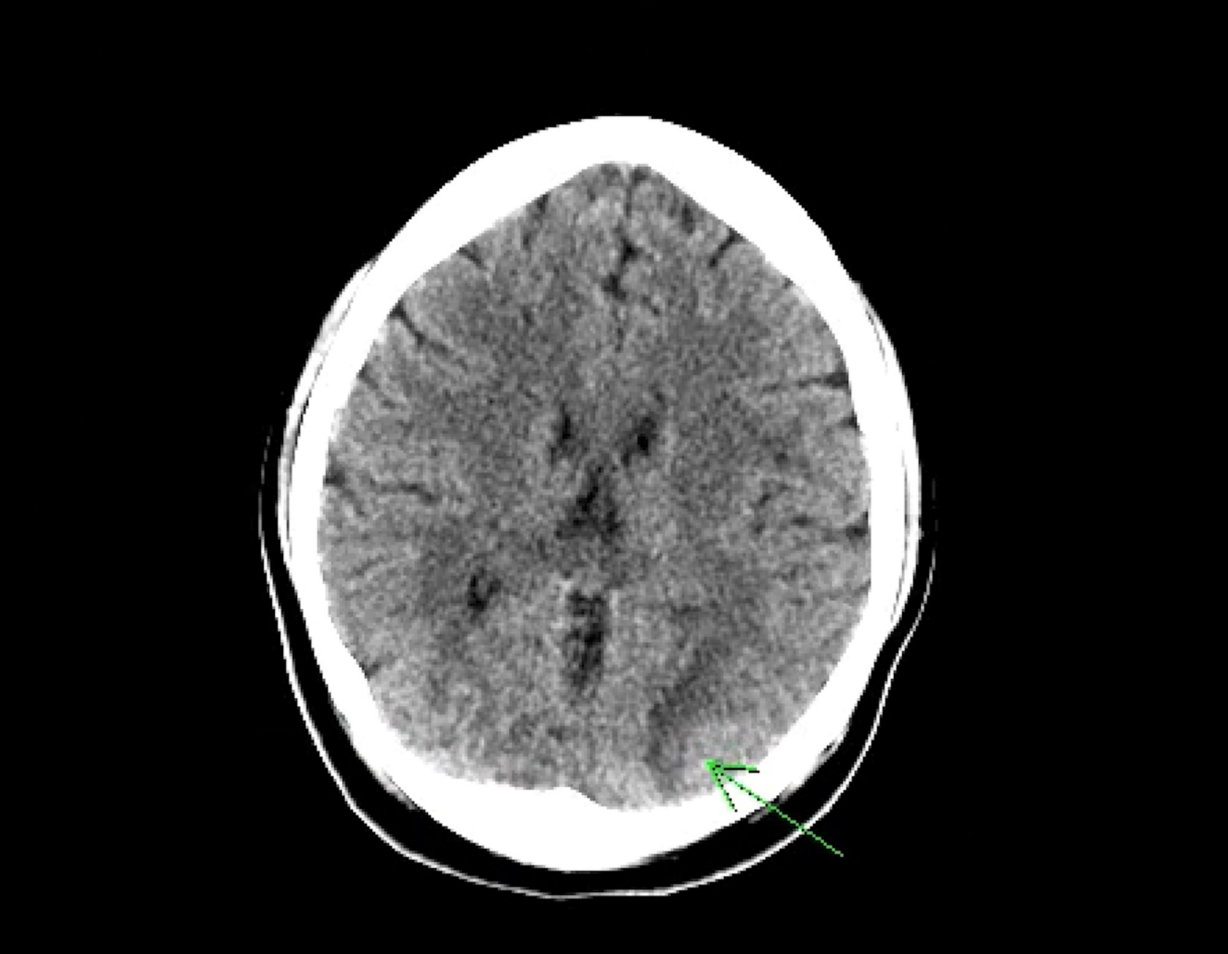
CT Head Without Contrast The left occipital lobe has low density noted with loss of gray-white differentiation concerning for possible acute infarction

**Figure 2:**
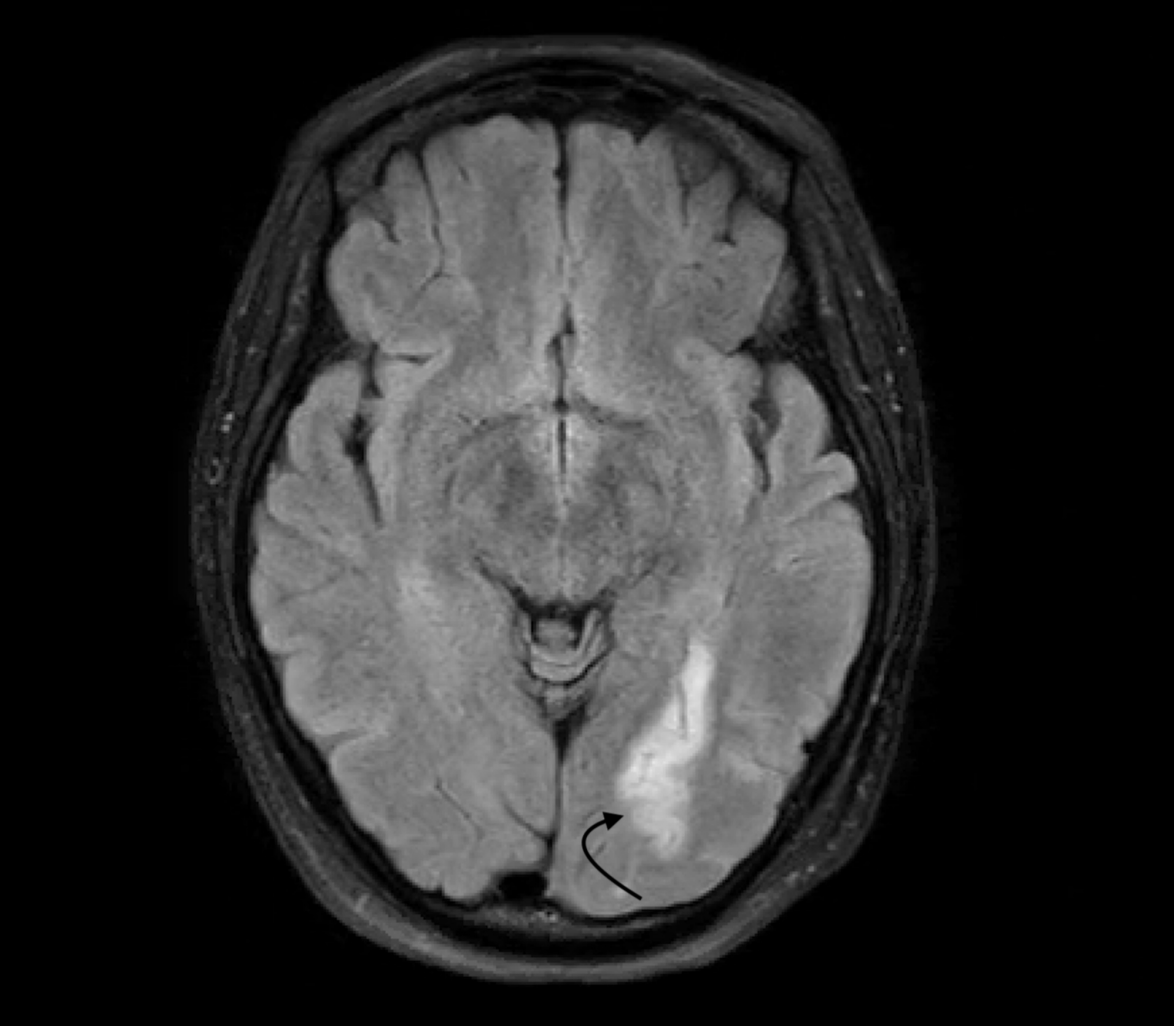
MRI of Brain With And Without Contrast An abnormal increase in signal density on FLAIR sequence consistent with an acute left posterior artery infarction

Her visual changes started when she was driving her car the day before admission that was accompanied by a throbbing headache along with photophobia, phonophobia which were not relieved by Ibuprofen or sleep. Her symptoms did not resolve after 12 hours; thus, she decided to present herself to the Emergency Department (ED) for further evaluation. In the ED, besides the complaints of blurry vision and headache, she denied any weakness, numbness, tingling, ataxia, similar prior episodes of visual changes, history of migraine, or alleviating factors. She noted persistent fatigue throughout this period but denied any dizziness, lightheadedness, shortness of breath, or chest pain. She denied any active bleeding on admission. The review of systems was otherwise negative. Her initial vital signs were: temperature of 99.6°F, blood pressure of 128/64 mmHg, respiratory rate 20 breaths per minute, heart rate 111 beats per minute, and oxygen saturation 99% on room air. Physical examination revealed a well-developed, well-nourished woman who was conversational, alert, and oriented. She had normal speech, language, funds of knowledge with intact recent and remote memory. Her visual field was normal by quadrant. Extraocular muscles were intact without gaze deviation, nystagmus, or ptosis. Pupils were equal and reactive to light. Normal motor strength and sensation were present throughout the body with negative Babinski sign. Cranial nerves 2-12 were intact. The rest of the physical examinations were unremarkable. Pertinent laboratory findings were hemoglobin 7.8 g/dL, hematocrit 26.1%, MCV 57.5 FL, MCHC 29.7%, RDW 17.2%, iron 11 ug/dL, iron-binding capacity 395 ug/gL, iron saturation 3%, ferritin <1 ng/mL, and platelet 364k/UL. The electrocardiogram showed normal sinus rhythm. CT head without contrast was concerned for a non-hemorrhagic left occipital lobe infarction in the posterior cerebral artery distribution [Figure 1]. CT angiographic examination demonstrated no focal high-grade intracranial stenosis, occlusion, or vasculitis. MRI of the brain revealed an abnormal increase in signal density on the FLAIR sequence that was consistent with an acute left posterior artery occlusion [Figure 2]. MRA of the brain and neck were subsequently unremarkable for any stenosis, occlusions, or dissections. Transthoracic echocardiogram revealed a small patent foramen ovale that was deemed to be clinically insignificant. She was treated with permissive hypertension, started on atorvastatin, clopidogrel without aspirin due to the history of gastric sleeve. She remained stable overnight.

On day 2 of hospitalization, her vital signs remained to be stable without new neurological findings on physical examination. Her hemoglobin level decreased from 7.8 g/dL to 6.8 g/dL. Later on during the day, the Rapid Response Team alert was activated due to an acute onset of left-sided numbness without other interval changes found on the rest of the neurological examination. She scored two on the National Institutes of Health Stroke Scale (NIHSS). CT scan of the head without contrast ruled out intracranial hemorrhage. MRI of the brain with and without contrast showed no interval changes to the left occipital lobe infarction. Repeat Hemoglobin was found to be 7.2 g/dL. It was then believed that the low hemoglobin level was the most likely cause of her acute ischemic stroke. She was treated with two units of packed red blood cell transfusion (PRBC) along with IV iron infusion with the goal of 1000 mg of the iron supplement per day for her underlying IDA. The patient reported an improvement of her symptoms a few hours later after the treatment began as her numbness and blurry vision both had resolved. She tolerated the blood transfusions well without complications.

There were no interval changes on day 3-7 of hospitalization. She was also started on daily folic acid. Atorvastatin was discontinued as hypercholesterolemia was the unlikely cause of her infarction given low LDL. Hemoglobin A1c was 5.4%. Hemoglobin levels remained to be greater than 8.5 g/dL status post the two units of PRBC transfusion. She had completed a total of five days course of iron transfusion. An intravaginal ultrasound was performed that did not reveal any endometrial abnormalities. Biopsy of the endometrium revealed benign proliferative endometrium, as well as a normal endocervix. The estrogen and progestin transdermal patches were discontinued as well. She was subsequently discharged home and was instructed to follow up with an outpatient hematologist for iron transfusion and hypercoagulable workup to assess whether it could be related to the CVA. Finally, she was also advised that the safest hormonal option would be a progesterone IUD for contraception as it has the least amount of systemic absorption.

She was followed closely by a hematologist in the outpatient setting. Hypercoagulable workup that included protein C activity, protein S antigen, PT/INR, PTT, peripheral blood smears, cardiolipin IgA, IgM, IgG, beta2-glycoprotein I IgA, IgM, IgG, factor V Leiden, prothrombin gene analysis was completely unremarkable. She also has been receiving intermittent iron transfusions, and her hemoglobin was closely monitored. Her hemoglobin levels were stable, with the most recent value of 12.0 g/dL. There were no reported residual neurological deficits.

## Discussion

Currently, there are no established explanations of why IDA causes strokes. However, IDA and CVA may have possible associations based on three mechanisms of action. The first hypothesis described that the decreased oxygen-carrying capacity in the blood from anemia might lead to low oxygen delivery to the brain, thus leading to ischemic injury, especially at the watershed regions. Another hypothesis stated that the low circulating oxygen levels might cause increased cerebral blood flow as a compensatory mechanism from the lack of oxygenation. This reaction subsequently increases turbulent flow, which damages the endothelium, leads to an abnormal platelet activation and aggregation sequence that ultimately forms a prothrombotic state and may subsequently cause distal clot embolization [2, 3]. This effect is more pronounced at the location of vessel bifurcations, such as the carotid bulb [5]. This mechanism of action was supported by research discoveries from Chang et al. which described how thrombotic and embolic ischemic strokes were associated with IDA [4]. The last hypothesis is that IDA induced microcytosis and thrombocytosis may trigger a hypercoagulable state and thrombosis. Low iron disinhibits megakaryocyte activity, thus resulting in a secondary thrombocytosis. Compensatory elevated erythropoietin levels from anemia also cause reactive thrombocytosis [6]. The decreased red cell deformability from microcytosis and thrombocytosis may lead to increased viscosity and formation of a hypercoagulable state [1, 7]. In addition, the reduced activity of iron-containing platelet monoamine oxidase, which catabolizes serotonin necessary for normal platelet function, can lead to increased platelet aggregation and thrombosis [6].

Keung et al. described two cases of reactive thrombocytosis as well as IDA associated pulmonary vascular and cerebrovascular thrombosis [8]. Iron is an important regulator of thrombopoiesis. However, a certain level of iron is needed for platelet production, and thus patients with very low iron levels may not have thrombocytosis [9]. Moreover, there is a weak correlation between platelet count and thrombosis as the platelet count does not always predict which patients are at a higher risk for thrombotic events with underlying essential thrombocythemia [1]. In contrast, abnormal platelet activation, as well as the platelet’s function, are more important factors than the absolute platelet count. Some case reports suggested that severe IDA even without reactive thrombocytosis could be associated with thrombotic events and thus a risk factor for ischemic stroke in young adults [6]. Moreover, anemia itself could be associated with stroke due to acute massive blood loss, causing systemic hypoperfusion with decreased cerebral blood flow and oxygen-carrying capacity, especially to watershed areas of the brain [10]. There was no evidence of thrombus formation, embolism, a large volume of blood loss or blood dyscrasia found in our patient. Despite the fact that atherosclerosis could still be considered as a risk factor of ischemic stroke in young adults, our patient had no cardiovascular risk factors as she had a normal body mass index without evidence of dyslipidemia, diabetes, or hypertension. She also had no clinical evidence of cocaine abuse, septic emboli, vasculitis, thrombophilic disorders including sickle cell disease (SCD) or inherited and acquired hypercoagulability state. Thus, the etiology of the acute occipital stroke in our patient is likely due to IDA that led to a decreased oxygen delivery to the brain.

A retrospective study by Maguire et al. discovered that children with stroke are eleven more times likely to have IDA than the control group [11]. Munot et al. described a case series study of four young children ranging 14 to 48 months old who developed ischemic stroke from either venous sinus thrombosis or arterial thrombus with significant underlying IDA [12]. Hartfield et al. published a case series of six children between 6-18 months of age with underlying IDA who were found to have ischemic stroke or venous thrombosis after the initial viral prodrome. However, the association between IDA and stroke has been less defined in adults compared to children [9]. Moreover, SCD is known to cause strokes in both children and adults, but the relationship between IDA and stroke in adults is currently not well explored [7]. Nationwide database survey from Taiwan by Chang et al. revealed that there is a significant association between IDA and ischemic stroke in adults as well as the importance of aggressive treatment of IDA [4].

In 1983, Alexander et al. first described a patient with IDA and thrombocytosis who developed right hemiparesis with aphasia that had improvement of neurological deficits one month after iron repletion [13]. There were fewer than twenty case reports of CVAs attributed by IDA. However, most of the cases were associated with either venous sinus thrombosis or carotid intraluminal thrombosis [1, 6, 8]. Akins et al. described three cases of women at respectively 20, 39, and 44 years old with IDA associated with stroke secondary to thrombocytosis and carotid artery thrombus. In contrast, Caglayan et al. described a 41-year-old woman with left frontal and bilateral parietal regions infarction secondary to IDA induced carotid thrombus [1, 5].

There are even fewer case reports of hypoxia-induced stroke without evidence of thrombosis [2, 3, 14]. Fluss et al. described a case of a 28-year-old man with underlying IDA who had periaqueductal gray matter infarction leading to esotropia but improved with emergent blood transfusion [2]. In contrast to our case, his hemoglobin was much lower at 1.12 g/dL on admission. Gopalratnam et al. described a similar case of a 20-year-old woman with a history of menorrhagia who was found to have bilateral cerebellar and left occipital lobe infarction secondary to IDA [3].

Previous case studies emphasized on the importance of early diagnosis of IDA along with its association with stroke. The studies also noted the importance of prompt treatment to prevent further complications from developing. One case report described a 47-year-old woman with right middle cerebral artery (MCA) infarction on admission was found to have a progression of the right MCA stroke after being found unresponsive with a new onset of left-sided weakness the next day. Her hemoglobin was 6.9 g/dL during this event, which was decreased from an initial value of 7.6 g/dL found on admission. Thus, it was hypothesized that she could have had a more favorable outcome if she were treated for IDA on admission that possibly could have lowered the chances of worsening stroke. Moreover, she had an improvement in her symptoms after receiving blood transfusion [14]. The presentation described in that study was very similar to our patient with the initial hemoglobin above 7.0 g/dL.

Naito et al. reported two cases of 42-year-old women with a history of menorrhagia due to uterine fibroids, who presented to the hospital with an acute ischemic stroke without thrombus. One of them had a confirmed diagnosis of IDA despite having a hemoglobin level of 11.6 g/dL. This case suggested that iron deficiency itself may cause stroke even without significant anemia [15]. In another case report by Sukdev et al., a 36-year-old woman with a history of diabetes mellitus was found to have diabetic ketoacidosis (DKA) and acute bilateral parieto-occipito-temporo-cerebellar infarction in the setting of severe IDA with the hemoglobin of 3.6 g/dL and ferritin level of 2.7 ng/mL. It was unclear whether the stroke was related to DKA or IDA. However, she had clinical improvements with no residual neurological deficits status post the improvement of the anemia. Thus, there could be a possible correlation between IDA treatment and stroke prognosis, as well as the potential outcome [16].

## Conclusions

IDA is a relatively common medical condition in young adults, especially in women of reproductive ages. One should consider IDA as a possible cause of ischemic strokes when other possibilities have been ruled out even though it might be extremely rare. Prompt diagnosis and treatment of IDA are essential to prevent not only CVA but also improve prognosis and outcome in patients who have suffered CVA secondary to IDA. Furthermore, the treatment of iron deficiency in young patients even without significant anemia may prevent thrombocytosis and platelet dysfunction, thus minimizing risk for thrombosis and its undesired complications.
